# Alcohol Consumption and Cardiovascular Disease Risk: Placing New Data in Context

**DOI:** 10.1007/s11883-022-00992-1

**Published:** 2022-02-07

**Authors:** Anna G. Hoek, Sabine van Oort, Kenneth J. Mukamal, Joline W. J. Beulens

**Affiliations:** 1 Amsterdam UMC, Vrije Universiteit Amsterdam, Department of Epidemiology & Data Science, Amsterdam Cardiovascular Sciences Research Institute, De Boelelaan 1117, Amsterdam, The Netherlands; 2 Beth Israel Deaconess Medical Center, Harvard Medical School and Harvard TH Chan School of Public Health, Boston, MA USA; 3 University Medical Centre Utrecht, Julius Center for Health Sciences and Primary Care, Utrecht University, Utrecht, The Netherlands

**Keywords:** Alcohol consumption, Cardiovascular disease, Moderate drinking

## Abstract

**Purpose of Review:**

A clear link between excessive alcohol consumption and cardiovascular disease (CVD) has been established, but no consensus exists on the effects of moderate alcohol consumption on CVD.

**Recent Findings:**

A lower risk of coronary heart disease and myocardial infarction among moderate drinkers compared to abstainers has been consistently observed in epidemiological studies and meta-analyses of these studies. However, ambiguity remains on the effect of alcohol on other CVDs and all-cause mortality. Short-term randomized controlled trials (RCT) have identified potentially beneficial effects of alcohol consumption on cardiovascular risk factors, but studies investigating genetic polymorphisms that influence alcohol consumption (i.e., Mendelian randomization) have yielded inconclusive results. To date, a long-term RCT providing causal evidence is lacking but urgently needed.

**Summary:**

Triangulation of evidence from different study designs, including long-term RCTs, pragmatic trials and the evaluation of policy measures, combined will lead to the best available evidence.

## Introduction

Alcohol consumption has been extensively studied as a modifiable risk factor for cardiovascular diseases (CVD). Excessive alcohol consumption (> 60 g/day in men and > 40 g/day in women) [1] is a well-known contributor to mortality and burden of CVDs [2, 3]. In contrast, a large number of observational studies report beneficial associations of low to moderate alcohol consumption (up to 60 g/day in men and up to 40 g/day in women) [1] with CVD. This results in a characteristic biphasic, “J-shaped” risk profile [4, 5••, 6] in which for low to moderate alcohol consumption, a lower CVD risk is observed compared to abstaining and excessive drinking. However, since most of the evidence of the protective effects of low to moderate alcohol consumption on CVD originates from observational studies, the question remains whether this effect is truly causal or merely a result of different forms of bias inherent to observational study designs [5••, 6]. To answer this question, there is an urgent need for studies assessing causality in the relationship between light to moderate alcohol consumption and CVDs. Unfortunately, to date, large-scale randomized controlled trials (RCT) with a sufficient follow-up period are lacking and previous efforts to conduct such studies have faced administrative and political challenges, despite intriguing evidence of feasibility [7–9].

Given the lack of an RCT that can serve as a gold standard, the focus in research has now shifted to new analytical methods and epidemiological designs such as pooled analyses from large consortia, instrumental variable analyses using genetic polymorphisms (e.g., Mendelian randomization (MR) studies), and RCTs investigating intermediate endpoints in the hope of shedding new light on the association between alcohol consumption and CVD. However, none of these studies has been able to resolve the pressing question of whether there is a true protective effect of moderate alcohol consumption. In addition, while such studies are innovative and potentially informative, they are not free of their own limitations and caution is equally required when interpreting the results of these new studies.

Our review aims to summarize previous efforts to investigate the relationship of alcohol consumption with CVD risk using classic observational epidemiologic designs, RCTs and MR studies. We will elaborate on the strengths and weaknesses of the different designs and offer new directions for research for the future.

## Evidence from Observational Research

Over the last five decades [10], hundreds and perhaps thousands of observational studies, systematic reviews and combined meta-analyses investigating the associations of alcohol consumption with CVD and mortality have been published. Observational studies have consistently but not invariably found that alcohol consumption above recommended limits is associated with a higher CVD incidence, cardiovascular and total mortality [2, 3]. However, more variable associations have been reported in studies investigating the impact of alcohol consumption within recommended limits on CVD incidence and mortality, compared to alcohol abstainers and excessive drinkers.

Early studies investigating alcohol consumption and cardiovascular health outcomes observed a “J”-shaped association, indicating a reduced risk for CVD incidence, specific and all-cause mortality for within-guideline drinkers compared to either abstainers or excessive drinkers. Protective associations have been best documented for myocardial infarction [11–14], but are also found when investigating heart failure [15, 16], peripheral artery disease [15], abdominal aortic aneurysm [15], hypertension [14], type 2 diabetes [17–19], (ischemic) stroke [14, 15, 20], cardiovascular mortality [15, 21] and all-cause mortality [12, 14]. Furthermore, these lower risks have been corroborated by meta-analyses [3, 4, 16, 22, 23]. Recently, several large studies and meta-analyses that used updated methodology, with the aim of minimizing the impact of bias inherent to observational study designs, have challenged the apparent association of limited alcohol consumption with lower cardiovascular risk [5••, 6, 24–26]. In a meta-analysis, Wood et al. analyzed data of 599,912 current drinkers without baseline CVD and found linear direct associations between alcohol consumption and risks of stroke, coronary disease, heart failure, fatal hypertensive disease and fatal aortic aneurysm, and inverse associations with overall cardiovascular disease and myocardial infarction for intake below 200 gm/week [5••]. A second meta-analysis executed by the Global Burden of Diseases, Injuries and Risk Factors Study (GBD) found similar results [6]. In both meta-analyses, no harmful or beneficial association of limited drinking with all-cause mortality was found, although Wood et al. observed lower risk among consumers of < 300 gm/week whose consumption was distributed over 3 or more days per week (i.e., within guidelines) [5••, 6]. The authors argue that the general assumption of a protective association of limited alcohol intake on CVD is flawed, and conclude that the relationship between alcohol consumption and CVD risk is complex and does not express itself as a single J-shaped association [5••]. Even though these studies consist of larger sample sizes and use updated methodology, they are not immune to selection bias and confounding inherent to observational research. Furthermore, combining multiple datasets into one mega cohort could actually cause data loss, therefore worsening these distorting factors and making outcomes less generalizable [27].

Even more recent observational studies have focused on specific niches of the relationship between alcohol consumption and CVD, testing the hypothesis that the relationship between alcohol and CVD does not fit into a one-size-fits-all approach and that the conflicting results found in previous studies might be due to differences within subpopulations. For example, investigators have observed apparent differences in the alcohol-CVD relationship between African-Americans, Asian-Americans and other race/ethnicity groups found [28–32] (perhaps related to well-known genetic differences in alcohol metabolism [33, 34]). Other recent papers have addressed drinking patterns, suggesting that regular drinking and drinking with meals may be associated with a lower risk of mortality [35–37] and beverage types, arguing for unique anti-oxidative and anti-inflammatory effects of red wine consumption [38]. However, although differences in subpopulations are present and should be taken into account in future guidelines, these studies have not resolved the question whether limited alcohol consumption is protective or harmful for the development of CVD.

## Methodological Problems Arising from Observational Research

When interpreting results from observational studies, several forms of bias should be considered. The first factor is the presence of uncontrolled confounding [39]. It is nearly impossible to account for all confounding factors in observational study designs, and this is likely to be particularly true for alcohol consumption, which has strong and varied determinants of exposure [25, 39–41]. A second caveat to consider is the “sick quitter” phenomenon, whereby abstainers (the referent category in many studies) include a mixture of long-term abstainers and those who have quit due to pre-existing illness. This results in an artificial elevation of the health risk among abstainers, in which it is not the absence of alcohol but impaired health status that increases the observed elevated risk [25, 41–50]. Even though the sick-quitter phenomenon can be partly solved by careful separation of abstainers category into former and never drinkers or by using rare alcohol intake as a reference category in analyses, it is hard to fully account for, and the use of rare drinkers poses its own problems due to underestimation.

A third problem in observational research is the use of questionnaires as a self-reported measurement to assess alcohol consumption, which is prone to systematic error due to recall bias [51, 52], underreporting [53], misreporting [53–55] and adherence to social and cultural norms in answering the questions [56, 57]. This can theoretically be overcome by using biomarkers of alcohol consumption as tool to assess habitual alcohol consumption [58]. However, most biomarkers for alcohol consumption are either not specific enough (e.g. liver function markers) or only capture recent consumption of the past days, often at high doses (e.g., ethyl glucuronide). Finally, the lack in uniformity on the definition of alcohol intake categories, the definition of abstinence and changes in drinking pattern over time are not standardized, resulting in heterogeneity of alcohol-related measurements, which makes it difficult to combine multiple datasets into larger analyses [53].

Although these forms of bias are inherent to observational studies, they do not diminish the importance of such studies to identify potential associations and they remain the only available approach to date to directly link alcohol consumption with long-term outcomes.

## Evidence from Randomized Controlled Trials

RCTs are not influenced by these forms of bias and are often regarded as the gold standard to prove causality in the relations between a risk factor and outcome. Due to the difficulty and costs of performing a long-term RCT of limited alcohol consumption on hard outcomes such as CVD, the main body of evidence comes from short-term RCTs on cardiovascular risk factors. Several meta-analyses show that moderate alcohol consumption increased high-density lipoprotein (HDL) cholesterol, apolipoprotein A1 and adiponectin [59•, 60, 61]. Moderate alcohol consumption has also shown to reduce low-density lipoprotein cholesterol, fibrinogen levels, Interleukin-6, HbA1c and fasting insulin concentrations in various studies [59•, 61]. No effects were observed on C-reactive protein or total cholesterol [59•, 61]. A meta-analysis of trials investigating the effect of a change of alcohol consumption on blood pressure showed that a reduction of alcohol consumption in those who drank two drinks/day or less was not effective, while this resulted in a significant reduction in systolic and diastolic blood pressure for heavier drinkers [62]. A meta-analysis of RCTs up to 2017 performed in people with diabetes showed no effect of moderate alcohol consumption during 4 to 104 weeks on HbA1c or blood glucose [63], although the longest such study to date identified a benefit specifically among those with slow ethanol metabolism [8]. Altogether, these studies provide plausible underlying mechanisms not only for the observed risk reduction of myocardial infarction with moderate alcohol consumption, but also for increased risks of other cardiovascular outcomes such as heart failure or stroke.

Despite the plausibility of this evidence, the duration of most RCTs has been 4 to 8 weeks, and effects over longer time periods on hard outcomes are therefore uncertain. Only a handful of RCTs have been performed with a duration of over 1 year. These RCTs, mainly conducted in high-risk populations with diabetes or previous myocardial infarction, showed improvements of HDL cholesterol, inflammatory markers and insulin sensitivity and even echocardiographically determined left ventricular function [9, 64], generally in line with shorter trials. The meta-analysis of RCTs on reduction of alcohol consumption on blood pressure included four trials with a duration of 1 year or longer, and these results were in line with the overall meta-analysis [62]. These longer trials suggest feasibility of a longer term RCT on hard outcomes to resolve the ongoing debate about effects of light to moderate alcohol consumption on CVD. Despite this, a recent effort to conduct such a long-term trial—the Moderate Alcohol and Cardiovascular Health trial (MACH15)—seemed feasible, but was terminated prematurely by the US National Institutes of Health, which funded the trial [7, 65].

## Executional Problems Regarding Randomized Controlled Trials

In contrast to the methodological problems faced when conducting and interpreting results from observational studies, important practical and ethical concerns face large-scale, long-term RCTs [7, 65]. Besides the question whether it is justifiable to impose alcohol consumption on individuals—although MACH15 was designed to exclude abstainers and heavy drinkers—one of the major concerns is on how the general public will conceive possible outcomes of RCTs. Since RCTs require strict inclusion and exclusion criteria, the results are by definition definitively applicable only to a selected part of the population. Because alcoholic beverages are widely consumed worldwide, the danger is that results of an RCT executed in a specific population could be wrongfully be applied to excluded individuals in whom the results would have differed [65]. Furthermore, several potentially adverse but rare outcomes, such as the risk of developing specific types of cancer, are impossible to investigate in an RCT due to the unrealistically large sample sizes needed. To date, all attempts to execute a large-scale RCT with sufficient follow-up time have stranded and currently no large RCTs are running to our knowledge.

## Evidence from Mendelian Randomization Studies

Facilitated by the availability of large genome-wide association studies (GWAS), MR has been increasingly used to investigate the relationship between alcohol consumption and CVD [66, 67•]. MR is a genetic instrumental variable analysis that uses genetic variants that are robustly associated with modifiable risk factors as instrumental variables [68]. Since genetic variants are randomly allocated at meiosis, they mimic an RCT setting in which all other variables except the exposure are distributed equally between subgroups. This theoretically makes the design less vulnerable to confounding and reverse causation bias [68], provided all instrumental variable assumptions hold.

For alcohol consumption and hard CVD endpoints, the majority of the MR studies thus far have reported null associations, but some have reported a higher risk of ischemic heart disease, peripheral artery disease, stroke and diabetes [67•]. For intermediate endpoints, evidence from MR studies has been more consistent, with the majority of the studies showing that genetically predicted higher alcohol consumption was associated with higher levels of blood pressure, triglycerides, HDL cholesterol, glucose and body mass index, and with lower levels of low-density lipoprotein cholesterol [67•]. The majority of methods used in MR assume linearity in the alcohol-outcome relationship, and are therefore not suitable to answer questions on relationships that are potentially non-linear, as could be the case with alcohol and CVD. Recently, new methodology has been developed to study potential non-linearity, such as the localized average causal effects (LACE) method [69], but its validity remains uncertain and, to date, only a few studies have used this method, with inconsistent results on the shape of the association [70, 71].

## Limitations of Using Mendelian Randomization Studies in Alcohol Research

MR studies allow for assessing causality when all assumptions are met, meaning the genetic variant (1) is robustly associated with the exposure, (2) is not associated with any confounder of the exposure outcome association and (3) only affects the outcome via its association with the exposure [27, 68, 72]. However, the use of SNPs related to alcohol consumption inherently poses a threat to these assumptions. Firstly, the explained variance for genetic instruments for alcohol consumption is generally low; therefore, large samples sizes are needed [67•]. Secondly, genetic instruments for alcohol consumption related to functional genes (e.g. ALDH2 and ADH1B/C) can be used in MR studies and explain a large part of the variance in Asian populations, but explain only a small part of the variance of alcohol consumption in non-Asian populations. Therefore, in non-Asian populations, other variants that are significantly associated with alcohol consumption, discovered through GWAS analyses, are commonly used. However, since the causality of these additional genes is not defined, it is difficult to assess whether the exposure gene is not associated with any confounder of the exposure-outcome association therefore possibly violating the second assumption [67•]. Thirdly, the most commonly used genetic variants for alcohol consumption share a similar pathway with variants associated with problematic alcohol consumption; therefore, the presence of pleiotropy is hard to avoid [73].

Furthermore, a few additional limitations are of special importance when using MR to investigate the role of alcohol consumption. Firstly, as mentioned above, to date, investigating potential non-linearity is difficult in such analyses and often not carried out. Furthermore, bias can be introduced in MR studies via assortative mating and dynastic effects [74, 75]. Assortative mating occurs if an individual with a particular genetic predisposition bases his partner selection on a certain genetically influenced phenotype. Dynastic effects are similar and occur when the expression of the parental genotype in the parental phenotype has a direct effect on their offspring’s phenotype. These are environmental and social factors that have the potential to affect the distribution of genetic variants for specific traits within the population. Studies of other exposures (e.g., BMI or education) have indeed shown that this leads to bias by inducing an association between the instrumental variable and the outcome [74, 75]. Although this has not been investigated for alcohol consumption, an earlier study provided evidence for presence of assortative mating for alcohol consumption [75], and parental substance use disorder is known to influence adult chronic diseases [76], suggesting dynastic effects. Finally, no evidence currently exists that genetic variants can separate the various domains of alcohol consumption (e.g., quantity versus frequency), despite their vastly different associations with CVD. Altogether, MR studies provide evidence from a different angle, but can by themselves not solve the debate on the role of limited drinking on cardiovascular health.

## A Century of Research on the Relationship of Alcohol and Cardiovascular Disease and Still No Consensus: How Do We Move Forward?

After nearly a century of research on the effects of alcohol consumption on cardiovascular health, we find ourselves running in circles, asking the same questions and reporting the same limitations. To move forward and gain greater insight into the health effects of limited alcohol consumption, a reconsideration of the standard of evidence is needed. Despite novel techniques to assess causality, a large-scale, long-term RCT still seems to be the only option to resolve the debate.

The design and initial conduct of the MACH15 trial show the feasibility of executing a large-scale trial. However, the conduct of such a trial itself became a matter of debate [65]. Although a trial of limited alcohol consumption could potentially show effects on CVD when using a high-risk population, it is unlikely that such a trial can definitively quantify effects on some adverse events such as breast cancer, simply because they are too rare (fortunately) and thus require astronomical sample sizes. Sample size calculations show that 60,000 individuals are needed to detect the expected risk of any alcohol-related cancer, and when aiming to investigate specific forms of cancer, such as breast cancer, up to five times bigger samples are needed [7]. Since in observational studies, limited alcohol consumption has no beneficial association with most cancers, a RCT specifically to prove alcohol causes cancer is ethically dubious. Secondly, the fear of falsely extrapolating results from a specific and high-risk study population to a more general public has been expressed. However, we argue that if a protective effect is observed in a high-risk population, these effects are likely to be physiologically generalizable to a lower risk population, albeit with a smaller absolute risk reduction. Most importantly, the fear of misinterpretation should not be a reason not to execute a RCT, but rather should motivate investigators to strive for excellent communication and prevent misinterpretation of the results [7]; fear of misinterpretation would otherwise derail virtually every important trial. Ultimately, we emphasize that alcohol is consumed by half of the world’s population, and to date, there is a nearly complete lack of causal evidence on its long-term effects. Therefore, obtaining highest level of evidence—in an appropriate way—is in everyone’s benefit. To argue otherwise is to leave patients, physicians and public health professionals in a state of artificially engineered ignorance.

**Fig. 1:**
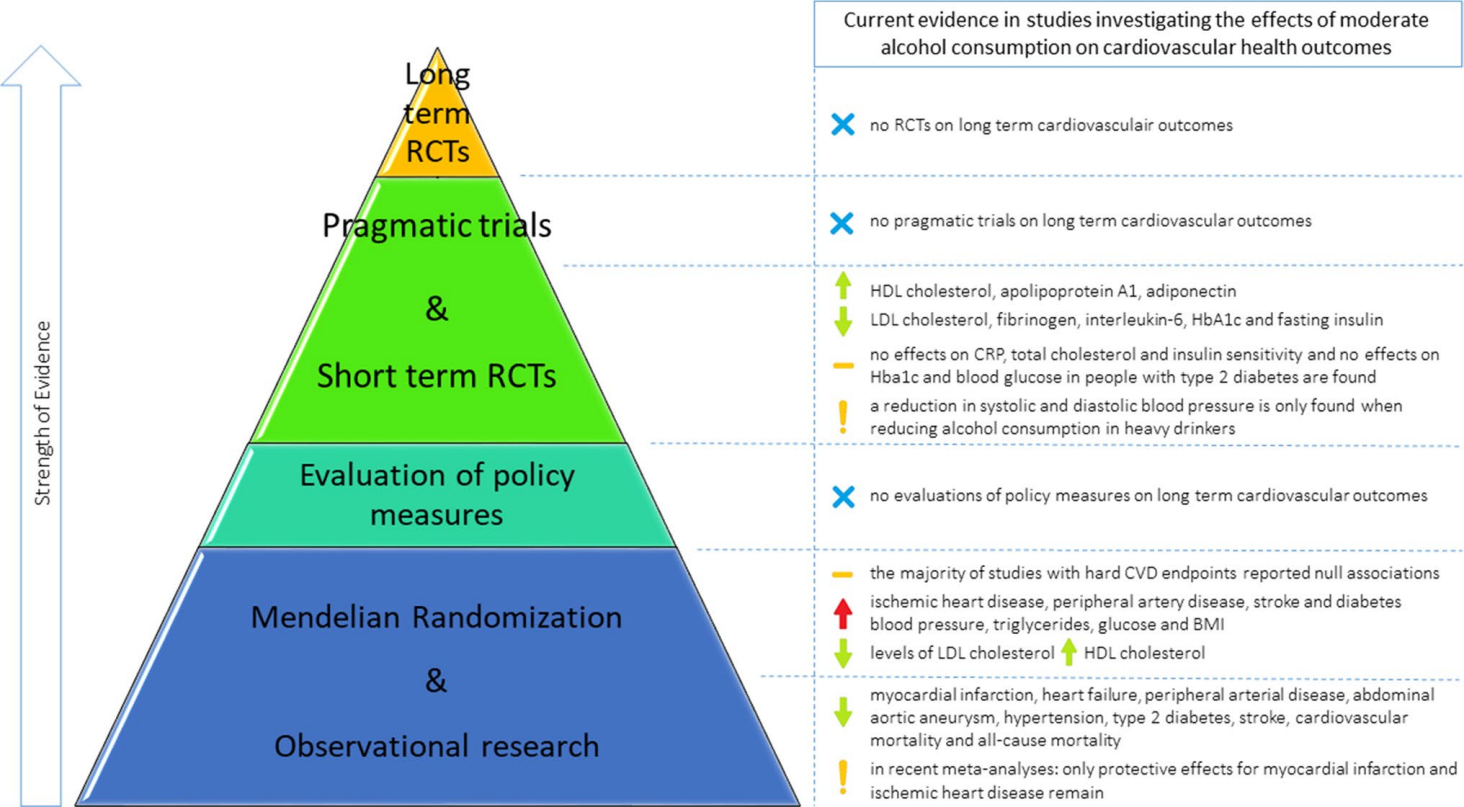
Overview of the current evidence of moderate alcohol consumption on cardiovascular health outcomes. RCTs: randomized controlled trials; HDL: high-density lipoprotein; LDL: low-density lipoprotein; HbA1C: hemoglobin A1C; BMI: body mass index

With no current RCTs running, it is likely that some time will pass before gold standard evidence is obtained. So, how do we move forward? The execution of a pragmatic trial, investigating the effects of lowering alcohol consumption on CVD endpoints, might be a solution. A pragmatic trial aims to evaluate whether a treatment works in daily clinical practice by using less controlled settings than when executing a classic RCT, but by still using randomization to compare different care strategies [77, 78]. This overcomes ethical concerns such as whether it is justifiable to impose people to drink alcohol, as in a pragmatic trial, the intervention could be to advice a reduction of the amount of alcohol already consumed. An advantage of this approach is the compatibility with the usual care situation, where a physician advises individuals to lower their alcohol consumption. Although these less controlled settings lead to the situation that the true biological “causal” effect cannot be estimated [79], it would provide more generalizable findings. Furthermore this design lends itself perfectly for stratification of the results in categories of alcohol consumption at baseline, which is insightful given the debate surrounding the possible J-shaped curve in the relationship of alcohol consumption on CVD outcomes. An alternative path to explore is the evaluation of the impact of alcohol consumption policy measures, in which pre- and post-intervention data in an interrupted time series analysis can be compared without using randomization [80, 81]. Alcohol consumption might lend itself particularly well for this kind of research, since the ambiguity on the relationship between limited alcohol consumption on health outcomes resulted in a large variety and frequent changes of alcohol consumption guidelines worldwide [82, 83]. Nonetheless, these studies too can be affected by confounding due to secular trends that co-occur with alcohol policy changes. Overall, we believe that the evidence from different approaches and study designs, with each their own strengths and limitations, when combined will result into the best available evidence [84, 85] (Fig. 1). Furthermore, to translate the research evidence to prevention in daily care, research on individual patient characteristics and absolute treatment effects is also needed. This can contribute to a tailored prevention approach for individual patients.

One pressing question remains to be answered: is there a safe drinking threshold for moderate amounts of alcohol consumption? Current guidelines generally recommend consumption of 1 drink or less daily (~ 14 g/day) for most individuals; some allow two drinks daily among younger and middle-aged men. In our opinion, these guidelines should be maintained until gold standard evidence is obtained, taking into account individual patient characteristics. And, most importantly, since worldwide alcohol consumption remains alarmingly high with an estimated consumption of 6.4 l of pure alcohol per capita [86], promoting adherence to these guidelines should remain a major public health priority.

## Conclusion

Controversy remains regarding the effects of moderate alcohol consumption on CVDs. A lower risk of coronary heart disease and myocardial infarction among moderate drinkers compared to abstainers has been reported in observational studies and was confirmed in the latest meta-analyses. However, on other cardiovascular outcomes and all-cause mortality, conflicting results have been reported. Many short-term RCTs and a few longer term trials have shown potentially beneficial effects of alcohol consumption on cardiovascular risk factors. However, MR studies investigating genetic polymorphisms that influence alcohol consumption often found non-protective effects, although results in MR studies are not always consistent and difficult to generalize (Figure 1).

Since alcohol is consumed by half of the world’s population, other approaches should be explored to define a safe limit to alcohol consumption. Future research should focus on executing pragmatic trials investigating the effects of lowering alcohol consumption in a daily clinical practice or on evaluating the impact of certain alcohol consumption regulating policy measures. Even though each of these designs have their own strengths and limitations, combined they can result in a careful triangulation of the evidence.
